# Scaffolds for the repair of bone defects in clinical studies: a systematic review

**DOI:** 10.1186/s13018-018-0724-2

**Published:** 2018-02-12

**Authors:** Jian-Hua Zeng, Shi-Wei Liu, Long Xiong, Peng Qiu, Ling-Hua Ding, Shi-Lang Xiong, Jing-Tang Li, Xin-Gen Liao, Zhi-Ming Tang

**Affiliations:** 1 Department of Orthopaedics, Jiangxi People’s Hospital, No.152, Ai guo Road, Nanchang, 330006 China; 2 Clinical Medicine, He University, Jinzhou, China; 3 Department of Orthopaedics, Jiangxi medical college, Nanchang university, Nanchang, China

**Keywords:** Scaffolds, Bone regeneration, Clinical

## Abstract

**Background:**

This systematic review aims to summarize the clinical studies on the use of scaffolds in the repair of bony defects.

**Methods:**

The relevant articles were searched through PubMed database. The following keywords and search terms were used: “scaffolds,” “patient,” “clinic,” “bone repair,” “bone regeneration,” “repairing bone defect,” “repair of bone,” “osteanagenesis,” “osteanaphysis,” and “osteoanagenesis.” The articles were screened according to inclusion and exclusion criteria, performed by two reviewers.

**Results:**

A total of 373 articles were obtained using PubMed database. After screening, 20 articles were identified as relevant for the purpose of this systematic review. We collected the data of biological scaffolds and synthetic scaffolds. There are eight clinical studies of biological scaffolds included collagen, gelatin, and cellular scaffolds for bone healing. In addition, 12 clinical studies of synthetic scaffolds on HAp, TCP, bonelike, and their complex scaffolds for repairing bone defects were involved in this systematic review.

**Conclusions:**

There are a lot of clinical evidences showed that application of scaffolds had a good ability to facilitate bone repair and osteogenesis. However, the ideal and reliable guidelines are insufficiently applied and the number and quality of studies in this field remain to be improved.

## Background

Healing of bone fractures and reconstruction of critical-sized bone defects represent a significant challenge. Autologous bone is the gold standard methods for the treatment of healing bone defects [1] due to stable structure, little immunogenicity [2], and natural osteogenic capacity [3–5]. However, the harvesting procedure has a high complication rate of 10–40%, including hemorrhage, nerve, and vascular lesions and postoperative pain [6]. Allograft bone, as bone graft substitute, shows good osteoconductive power and biomechanical characteristics and especially avoids the occurrence of complications [7]. However, the amount and quality of bone that can be harvested is limited, which restricts its use in large defects [8]. The disadvantages of bone autograft and allograft implantation have necessitated the development of alternative methods for bone repair [9].

A series of bone repair and transplantation substitutes have been derived with the development of material science and technology. In the past decades, cell- and gene-activating material, also known as bone-tissue engineering material, is the third generation bone-repair material. Tissue engineering material has been made into the extracellular matrix scaffold. The progenitor cell can proliferate and differentiate along scaffolds for better imitating the living situation of the surrounding tissue [10]. Tissue engineering scaffolds for bone regeneration have desirable characteristics of biocompatibility, non-toxicity, low cost, and non-carcinogenicity, with excellent osteoconductive and osteoinductive properties [11].

Biological scaffolds include corals, natural polymers, and demineralized bone matrix such as collagen sponge, gel foam, and cellular scaffold. Synthetic scaffolds include porous metals, synthetic polymers, and calcium phosphates (CaPs). Collagen contributes to mineral deposition, vascular ingrowth, and growth factor for bone regeneration [12]. CaPs ceramics is one of the most popular bone substitutes because its chemical composition resembles to bone mineral [13–15]. This feature enhances appropriate vascularization and stem cell proliferation and guides bone regeneration without causing any local or systemic toxicity [11]. Among the CaPs materials, hydroxyapatite (HAp) and β-tricalcium phosphate (β-TCP) are ideal substrates due to their excellent osteoconductive properties [16, 17].

Currently, one of the most advanced methods in tissue engineering is to transplant porous scaffolds with cell- and bone-stimulating agents into patients to form a complete bone transplanting. Tissue engineering scaffolds with osteoinductor were utilized for better bone regeneration by inducing bone cells to adhesion and proliferation. Mesenchymal stem cells (MSCs) can be well described and standardized, osteogenic differentiation from which is spontaneously into osteoblasts in vitro when compared to other mesenchyme tissues [18]. Bone morphogenetic protein (BMP), which combined with extracellular receptor, ultimately promote gene expression and induce mesenchymal stem cells to differentiate into osteoblasts [19, 20]. In addition, they enhance bone collagen synthesis and stimulate adjacent bone cells to grow [21, 22]. The periosteum is highly vascularized which can provide the cortical blood supply [23–25] and has been demonstrated to be an important factor in healing long bone fractures [26, 27].

To our knowledge, there have been several systematic reviews of scaffold materials, animal study, preclinical study, and carrier in MSCs for bone repair [11, 12, 28–30]. While little systematic review of bone-repair scaffolds were related to the clinical application. To our knowledge, this is the first report of a systematic review regarding on the clinical studies for scaffolds of bone defects. Therefore, the main aim of this study was to examine and summarize clinical studies on the use of scaffolds in the treatment of bony defects.

## Methods

The relevant articles were searched through PubMed database. The following keywords and search terms were used: “scaffolds,” “patient,” “clinic,” “bone repair,” “bone regeneration,” “repairing bone defect,” “repair of bone,” “osteanagenesis,” “osteanaphysis,” and “osteoanagenesis.” The articles were screened according to inclusion and exclusion criteria, performed by two reviewers.

Search terms were selected according to guidelines on Table 1.

**Table 1:** Search strategy used in PubMed

Search terms	
#4 Search ((#1) AND #2) AND #3 #3 Search “patient” OR “clinic” #2 Search (“bone repair” OR “bone regeneration” OR “repairing bone defect” OR “repair of bone” OR “osteanagenesis” OR “osteanaphysis” OR “osteoanagenesis”) #1 Search “Scaffold*”	

Inclusion criteria

1. Studies on scaffolds used in bone repair and bone regeneration
2. Clinical studies

Exclusion criteria

1. Studies that used scaffolds in engineering of cartilage
2. Studies in the field of maxillofacial or neurosurgical defects
3. Studies that used scaffolds in the treatment of periodontal and alveolar defects
4. Studies only in vitro
5. Animals studies
6. Articles in any language other than English
7. Unpublished literature

Any dispute about whether an article fits the inclusion criteria, such as study type, scaffold function, treatment efficacy, and safety, was resolved by discussion.

## Results

**Fig. 1:**
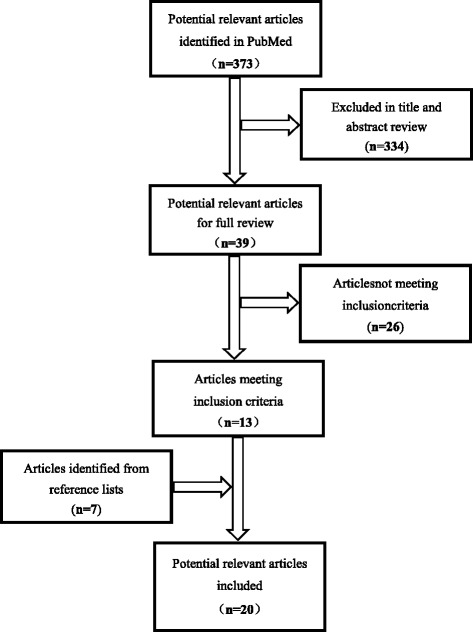
The flow chart of literature selection for the systematic review

A total of 373 articles were reviewed, and 20 articles were identified as relevant for the purpose of this systematic literature review. The studies included have been summarized in Fig. 1. There are eight clinical trials on the use of biological scaffolds including collagen scaffolds, complex cellular scaffolds, and gel foam scaffolds in Table 2. Biological scaffolds usually have good osteogenesis, biocompatibility, and security. Four studies assessed the use of collagen bone scaffolds with osteoinductor [18–20, 31], which is performed by Calori et al. [31], and compared the efficacy of recombinant bone morphogenetic protein 7 (rhBMP-7) and platelet-rich plasma (PRP) (both in collagen scaffolds) in the treatment of persistent fracture non-unions in 120 cases. A lower median clinical and radiographic healing time were observed in the rhBMP-7 group than the PRP group. Jager et al. [18] treated ten patients with volumetric bone deficiencies in a study that used porous collagen I as a scaffold with MSCs and bone marrow aspirate in a 3-year follow-up. The remaining two studies [19, 20] evaluated the safety and efficacy of the use of an absorbable collagen sponge impregnated with recombinant bone morphogenetic protein (rhBMP-2). The study demonstrated that rhBMP-2 is a safe bone-stimulating agent, which can significantly reduce the frequency of bone-grafting procedures for the treatment of type-III open tibial fractures. Jager et al. [32] investigated the potency of bone marrow aspiration concentrate (BMAC) to augment bone grafting and support bone healing in 39 patients of volumetric bone deficiencies. The result showed that all patients appeared new bone formation in radiographs during follow-up. Two studies on clinic involved with cellular scaffolds [33, 34]. Cuthbert et al. [33] reported that the complex cellular scaffolds with induced membrane (IM) were used for treating critical size defects of eight patients. They concluded that the constitution of IM like periosteum and had a cellular composition and molecular profile, which facilitated large defect repair. Another study [34] evaluated new bone formation after the application of BMAC and recorded possible complications in 101 bone defect patients. The majority of patients were not observed to have infections, excessive new bone formation, and induction of tumor formation, morbidity, and complications within the 24-month follow-up period. Philip et al. [35] showed that majority of ribs treated with gel foam scaffolds re-grew to normal morphology within 3–6 months of costectomy compared to those without scaffold. Although biological scaffolds have good bone formation performance, the weak mechanical strength is the main reason for not as a solo scaffold.

**Table 2:** Biological scaffolds

Scaffold	Case	Duration	Trial	Results	Complications	Reference
Collagen scaffold	Long bone non-unions (group rhBMP-7 *n* = 60 group PRP) *n* = 60)	> 9 months	The purpose of this prospective randomized clinical study was to compare the efficacy of rhBMP-7 and PRP as bone-stimulating agents in the treatment of persistent fracture non-unions.	Both clinical and radiological union occurred in 52 (86.7%) cases of the rhBMP-7 group compared to 41 (68.3%) cases of the PRP group, with a lower median clinical and radiographic healing time observed in the rhBMP-7 group.	Complications including severe, moderate and mild; adverse events were classified as serious or non-serious	Calori et al. [31]
Porous collagen I scaffold	Volumetric bone deficiencies (*n* = 10)	3 years	The clinical outcomes of ten patients with volumetric bone deficiencies treated with MSCs and bone marrow aspirate are presented in this case series. Results were evaluated with radiographs.	All patients showed bony healing and/or sufficient new bone formation within follow-up. There were no restrictions to any physical activities prior to the causative disease at latest follow-up. All patients returned to their profession after treatment.	1 prolonged hematoma	Jager et al. [18]
Absorbable collagen sponge scaffold	Open tibial fractures (*n* = 450)	12 months	The objective of this study was to evaluate the safety and efficacy of the use of rhBMP-2. 450 patients with an open tibial fracture were randomized to receive either the standard of care, the standard of care and an implant containing 0.75 mg/mL of rhBMP-2, or the standard of care and an implant containing 1.50 mg/mL of rhBMP-2. The rhBMP-2 implant (rhBMP-2 applied to an absorbable collagen sponge) was placed over the fracture at the time of definitive wound closure.	The rhBMP-2 implant was safe and, when 1.50 mg/mL was used, significantly superior to the standard of care in reducing the frequency of secondary interventions and the overall invasiveness of the procedures, accelerating fracture and wound-healing, and reducing the infection rate in patients with an open fracture of the tibia.	Local adverse events including inflammation, infection, hardware failure, pain, and complications.	Govender et al. [19]
Absorbable collagen sponge scaffold	Open tibial fractures (*n* = 510)	12 months	The objective of the current study was to perform a subgroup analysis of the combined data from these studies. 510 patients were randomized to receive the control treatment or the control treatment and an absorbable collagen sponge impregnated with one of two concentrations of rhBMP-2. The rhBMP-2 implant was placed over the fracture at the time of definitive wound closure.	The addition of rhBMP-2 to the treatment of type-III open tibial fractures can significantly reduce the frequency of bone-grafting procedures and other secondary interventions. This analysis establishes the clinical efficacy of rhBMP-2 combined with an absorbable collagen sponge implant for the treatment of these severe fractures	NC	Swiontkowski et al. [20]
Collagen sponge and Hap scaffold	Volumetric bone deficiencies patients (*n* = 39) with collagen scaffold (*n* = 12) and HA scaffold(*n* = 27)	> 6 months	The study investigated the potency of BMAC to augment bone grafting and support bone healing. The functional and radiographic outcome of 39 patients with treated with BMAC are presented and evaluated	All patients showed new bone formation in radiographs during follow-up. Two patients underwent revision surgery due to a lack in bone healing. The postoperative bone formation and complete bone healing appeared earlier in the HA group in contrast to the Collagen group.	1 persisting hematoma, 3 wound secretions	Jager et al. [32]
Gel foam scaffold	16 patients (*n* = 51 ribs) who underwent costectomy with gel foam scaffold and 15 patients (*n* = 33 ribs) with no scaffold.	6 months	The aim of the study is to compare rib regeneration with a scaffold placed intra-periosteally against no scaffold, after costectomy in adolescent idiopathic scoliosis. Patients were analyzed radiographically for rib regeneration and morphology.	The resulting data showed that majority of ribs re-grew to normal morphology in 3–6 months in the trial group. Ribs treated by placement of gel foam scaffold regenerate to a near normal radiological profile within 6 months of costectomy compared to a slower regeneration in those without gel foam scaffold.	NC	Philip et al. [35]
Complex cellular scaffold	Post-traumatic nature bone defects (*n* = 8)	6–8 weeks	Critical size defects were treated with the IM technique. Morphological characteristics, cell composition, and growth factor expression were compared with healthy diaphyseal P. Functional and molecular evaluation of MSC activity was performed.	Both tissues shared similar morphology although IM was significantly thicker than P. The IM resembles periosteum with a cellular composition and molecular profile facilitating large defect repair and therefore may be described as an “induced-periosteum”	NC	Cuthbert et al. [33]
Intra-operative cellular bone substitution material scaffold	Various bone healing disturbances (*n* = 101)	2-24 months	The objective of the study was to evaluate new bone formation after the application of BMAC as well as to record complications. The application of BMAC was performed via a local injection as part of a core decompression (*n* = 72) or by the local adsorption of intra-operative cellular bone substitution material (scaffold) incubated with BMAC during osteosynthesis (*n* = 17)or in further surgery (*n* = 12).	Only 2 patients were observed complications. Infections, excessive new bone formation, induction of tumor formation and morbidity due to the bone marrow aspiration from the iliac crest were not seen.	2 complications	Hendrich et al. [34]

**Table 3:** Synthetic scaffolds

Scaffold	Case (*n* = sample)	Duration	Trial	Results	Complications	Reference
HAp	Bone tumors (*n* = 3)	29–43 months	MSCs obtained from each patient’s bone marrow cells were forced to differentiate into osteoblasts followed by bone matrix formation on HAp ceramics to heal bone tumors using tissue-engineered implants. Serial plain radiographs and computed tomography images were used to observe results.	The strong osteogenic ability of the implants, as evidenced by high osteoblastic activity, was confirmed. The tissue-engineered HAp was used to fill the patient’s bone cavity after tumor curettage. Immediate healing potential was found and no adverse reactions were noted in these patients	NC	Morishita et al. [36]
Porous HAp ceramic scaffold	Large bone diaphysis defects (*n* = 4)	6.5 years	Cells from the patients’ bone marrow stroma were expanded in culture and seeded onto porous HAp ceramic scaffolds designed to match the bone deficit in terms of size and shape. Conventional radiographs and CT scans evaluated patients.	No major complications occurred in the early or late postoperative periods. No signs of pain, swelling, or infection were observed at the implantation site. Complete fusion between the implant and the host bone occurred 5 to 7 months after surgery.	No major complications and No signs of pain, swelling, or infection.	Marcacci et al. [37]
HAp	Large bone defects (*n* = 3)	> 15 months	Osteoprogenitor cells were isolated from bone marrow and expanded ex vivo. These cells were placed on macroporous hydroxyapatite scaffolds and implanted at the lesion sites. External fixation was provided initially for mechanical stability and was subsequently removed.	In all three patients, radiographs and computed tomographic scans revealed abundant callus formation along the implants and good integration at the interfaces with the host bones by the second month after surgery.	NC	Quarto et al. [38]
IP-CHA	22 patients (*n* = 30 hips) who used BMMNCs with IP-CHA and 8 patients (*n* = 9 hips) with cell-free IP-CHA of osteonecrosis of the femoral head	> 12 months	We have investigated the effectiveness of the transplantation of BMMNCs and cell-free with IP-CHA on early bone repair for osteonecrosis of the femoral head.	In the BMMNC group, a reduction in the size of the osteonecrotic lesion was observed subsequent to hypertrophy of the bone in the transition zone and three patients were detected extensive collapse. In the control group, severe collapse of the femoral head occurred in six of eight hips.	No intra- or post-operative complications	Yamasaki et al. [39]
HAp/type I collagen composite scaffold	Bone defects by benign bone tumors with HAp/Col (*n* = 63) and β-TCP (*n* = 63)	18 and 24 weeks	The efficacy and safety of HAp/Col were assessed in comparison β-TCP. X-ray images and blood tests and observation of the surgical site were performed to evaluate the efficacy and safety of the implants.	The highest grade of bone regeneration was more frequent in the porous HAp/Col group than in the porous β-TCP group (*p* = 0.0004 and 0.0254 respectively). The incidence of adverse effects was higher in the porous HAp/Col group than in the β-TCP group.	NC	Sotome et al. [40]
HAp/TCP scaffold	Spondylolisthesis (*n* = 25)	12–27 months	Autograft/ TSRH pedicle screw instrumentation (*n* = 5), rhBMP-2/TSRH (*n* = 11), and rhBMP-2 only without internal fixation (*n* = 9). On each side, 20 mg of rhBMP-2 was delivered on a carrier consisting of 60% HAp and 40% TCP granules (10 cm^3^/side).	RhBMP-2 with the biphasic CaPs granules induced radiographic posterolateral lumbar spine fusion with or without internal fixation in patients whose spondylolisthesis did not exceed grade 1. Statistically greater and quicker improvement in patient-derived clinical outcome was measured in the rhBMP-2 groups.	No complications	Boden et al. [41]
rCPBS scaffold	Recalcitrant tibial fracture nonunion (*n* = 20)	14 ± 2.7 months	All patients were treated with a procedure including debridement and decortications of the bone ends, nonunion fixation with a locking plate, and filling of the bony defect with a combined graft of rhBMP-7 (as osteoinductor) with an rCPBS (as scaffold)	No specific complication of rCPBS or rhBMP-7 was encountered. The application of rCPBS combined with rhBMP-7, without any bone grafting, is safe and efficient in the treatment of recalcitrant bone union.	No specific complication	Ollivier et al. [43]
β-TCP scaffold	Femoral defects with autologous MSC/ β-TCP (*n* = 9) and β-TCP (*n* = 9)	12 months	Compare healing quality of implantation into femoral defects during revision total hip arthroplasty, containing either expanded autologous MSC (trial group) or Β-phosphate alone (control group).	A significant difference in the bone defect healing was observed between both groups of patients (*p* < 0.05). Trabecular remodeling was found in all nine patients in the trial group, and only 1 patient in the control group.	2 dislocation and 1 pulmonary embolism, and 1 cardiac arrhythmia	Sponer et al. [42]
Bonelike scaffold	Medial compartment osteoarthritis of the knee (*n* = 11)	12 months	The aim of the present work was to assess the biological behavior of Bonelike graft and osteoconductive properties and resorption characteristics of the granulesin. Radiological follow-up, scanning electron microscopy, histological analysis and histomorphometric measurements were conducted on the retrieved samples to assess bone regeneration in the defect area.	Bonelike acted as an excellent bioactive scaffold, allowing the migration, proliferation, and differentiation of bone cells on its surface, and therefore regeneration of the defects was achieved in a rapid, controlled manner.	NC	Gutierres et al. [47]
BoneSave (TCP/HAp)	Posterolateral inter-transverse spinal defects(*n* = 45)	46 months	Analogue scales for pain, patient global impression of change, work status, persisting symptoms and patient satisfaction data, radiological evaluation of fusion was carried out from the most recent spinal radiographs available for each patient	Significant post-operative improvements were seen across all outcome measures in the large majority of cases. Successful fusion was achieved in 56.7% of cases.	Avoid donor site morbidity	Kapur et al. [44]
BoneSave (TCP/HAp)	34 patients received uncemented acetabular components (*n* = 34) and 9 received cemented components (*n* = 9)	2 years	BoneSave using mixtures of allograft and BoneSave in impaction grafting were used to assess the effectiveness.	There were no re-revisions and there was no implant migration. Complications were rare (1 fracture, 2 dislocations). Impaction grafting of BoneSave and allograft is an effective method of dealing with loss of bone stock at revision hip surgery in short-term study.	1 fracture, 2 dislocations	Blom et al. [46]
BoneSave (TCP/HAp)	34 patients received uncemented acetabular components (*n* = 34) and 9 received cemented components (*n* = 9)	7 years	Patients were followed up radiographically and with the SAPS, OHS, and SF12 health survey. Kaplan-Meier survivorship analysis was performed with revision of the acetabular component, revision of any part of the construct, and reoperation as endpoints.	1 patient had been revised for aseptic loosening of the acetabulum and 1 for deep infection. BoneSave is a reliable material for impaction grafting of the acetabulum when used in conjunction with femoral head allograft in medium-term study.	NC	Whitehouse et al. [45]

Therefore, due to the above reason, synthetic scaffolds of tissue engineering materials are used comprehensively, which performed good property of new bone formation and mechanical strength. The uses of synthetic scaffolds examined in clinical studies are summarized in Table 3. HAp, β-TCP, and their complex materials with bone-stimulating agents were used in the most of synthetic scaffolds. Six studies investigated the use of HAp and its complex scaffolds in bone defects. Morishita et al. [36] reported strong osteogenic ability of HAp scaffolds with MSCs after tumor curettage and found no adverse reactions in all three patients. Cells were isolated from bone marrow and seeded onto the porous HAp scaffolds in two related studies [37, 38]. Both studies showed abundant cellar formation along the implants after several months. Furthermore, Marcacci et al. [37] found no signs of pain, swelling, or infection at the implantation site and no major complications in the early or late postoperative periods. Yamasaki et al. [39] compared the effectiveness of the transplantation of bone-marrow-derived mononuclear cells (BMMNCs) plus interconnected porous calcium hydroxyapatite (IP-CHA) on early bone repair for osteonecrosis of the femoral head with those of without BMMNCs and found that the implantation of BMMNCs and IP-CHA appears to confer benefit in the repair of osteonecrosis and in the prevention of collapse. Sotome et al. [40] assessed the efficacy and safety of HAp/collagen scaffold in comparison to β-TCP and showed the porous HAp/collagen group had the highest grade of bone regeneration but also associated with higher incidence of adverse effects. The use of rhBMP-2 in the biphasic CaPs granules with or without internal fixation in patients of spondylolisthesis did not exceed grade 1 in Boden et al.’s study. However, statistically greater and quicker improvement in patient-derived clinical outcome was measured in the rhBMP-2 groups [41]. Five studies examined the use of β-TCP as a fundamental material and composition to manage bone defects in clinical studies. One study [42] combined a β-TCP scaffold with MSCs and showed that the addition of MSCs resulted in more trabecular remodeling in femoral defects. Ollivier et al. [43] showed that the addition of rhBMP-7 to a TCP scaffold is safe and efficient in the treatment of recalcitrant bone union. Three studies [44–46] in clinical studies examined the use of BoneSave, a porous bone graft substitute made of β-TCP and HAp ceramic. Kapur et al. [44] showed that 56.7% of cases achieved successful fusion in 45 posterolateral inter-transverse spinal patients. Two of studies involved impaction grafting of BoneSave and allograft, which is an effective method of dealing with loss of the acetabulum in short- and medium-term studies [45, 46]. A novel study about bonelike scaffold was studied [47]. The result indicated that bonelike can be an excellent bioactive scaffold and therefore regeneration of the defects was achieved in a rapid, controlled manner.

## Discussion

In this systematic review, 4 studies of femoral or acetabular defects, 3 studies of tibial fractures, 2 studies of large bone defects, 2 bone tumors studies, 2 studies of spinal defects, 2 volumetric bone deficiencies studies, 1 long bone defect study, 1ribs study, 1 study of knees, 1 post-traumatic bone defects study, 1 various bone study were included in the systematic review. The common defect position and the important bone types were involved in this systematic review. All the mentioned results of studies achieved a favorable efficacy of bone regeneration and an increased heal rate of bone defects, which demonstrated the scaffolds for bone repair played a critical role of bone heal.

As we know, the complications in scaffold of bone regeneration fields are an important challenge for the orthopedic surgery because infectious complications are major threat to the process of patient recovery. Complex methods and long-term process were required especially for effective antibiotic therapy which is a foundation of therapy. In our research, we added the information related to complications and adverse event of clinical studies in Tables 2 and 3. Among the 8 studies of biological scaffolds, five studies presented the data of postoperative complication and adverse effect. Therein, major complications such as fracture, hematoma, pain, inflammation, and infection were the main reasons affecting the progress of postoperative recover. Among the 12 studies of synthetic scaffolds, seven mentioned these postoperative results of complication and adverse event. Five of the seven studies on complications indicated that there were no major postoperative complications and no signs of infections. Another two researches of the seven reported only several cases had complications including dislocation, pulmonary embolism, and fracture. In the BoneSave substitute, the common complications of donor site morbidity were involved in these studies. In general, these results demonstrated that the complications discovered in synthetic scaffolds were less than those of the biological scaffolds. This may be due to relatively poor antibacterial property and bio-compatibility of biological scaffolds.

Current autografts and allografts are considered as the gold standard treatment for bone defects and mostly harvested from the iliac crest. However, the disadvantages of donor site morbidity, disease transmission, and susceptible to infection limit its application. Therefore, tissue-engineered grafts had been driven to the investigation and development of synthetic and biological bone-tissue engineering applications. The third bone grafting material, which is the mixture of scaffolds, cell- and gene-activating grafts, is the new biological bone repair material.

An ideal biomaterial should stimulate or induce the differentiation and proliferation of stem cells and osteoblast cells to heal defect sites [30, 48]. In eight clinical studies of biological scaffolds, collagen, gel, and cellular scaffolds for bone healing were included in the review. Collagen is a natural polymer for biomedical application with resorbable properties [48] and showed sufficient osteanagenesis [18–20, 31]. Gelatin has many advantages that included biocompatibility, biodegradability, cost effectiveness, common availability, and more accessible functional groups, making it a suitable material for bone tissue applications [49]. The utility of cellular scaffolds also facilitated bone defect repair [33, 34]. Most of biological materials tend to have weak mechanical strength, so it is rarely used as a single bone regeneration scaffold in tissue engineering and usually combined with other materials of good mechanical strength for repairing bone defects. The composite scaffold of HAp/collagen showed the highest grade of bone regeneration [40].

Twelve clinical studies on synthetic scaffolds were involved with HAp, TCP, and their complexes for repairing bone defects. HAp is the most important inorganic component of bone tissue with widely available bioactive and bioresorbable trait [7]. Four studies [36–39] pointed out that HAp had strong osteogenic ability for bone healing, and adverse reaction and major complications were not seen. The BoneSave, a matrix of HAp and β-TCP, are ideal biphasic porous ceramic bone graft substitutes due to their excellent osseointegration properties, but concerns have been raised as to their ability to maintain their structural integrity under load [44–46].

Furthermore, several tissue engineering materials such as collagen I, TCP, or HAp are currently available clinically as bone substitutes and can be used as scaffolds in combination with the bone-stimulating agents to expedite bone healing. MSCs can be spontaneous differentiation into osteoblasts. The discovery of BMPs appears to be the most selective for expedite gene expression and osteoblasts differentiation [50]. Among this, rhBMP-2 and rhBMP-7 are used in a variety of complex orthopedic conditions. In several clinical studies [18, 19, 31, 41, 43], BMPs had the greatest efficacy as bone-stimulating agents for bone defects treatment. The periosteum provides the cortical blood supply in healing critical size defects. The technology of induced membrane (IM) serves as a conduit to contain cells or bone graft for bone regeneration [33].

Most of the systematic reviews in bone repair are related to animal experiments or preclinical trials. There is almost no systematic review for the clinical application of bone-repair scaffolds. In Crowley et al.’s review [12], only five studies about scaffolds for bone regeneration are related to clinical trials. Therein, three of these studies related to small numbers and four of the studies had no control group and all of the studies involved short follow-up time of several months and even weeks. In summary, it lacks of representative and convincing to demonstrate the clinical studies of bone-repair scaffolds. However, all the 20 articles included in our review were related to the clinical study of scaffolds for bone repair. Only four studies used small samples less than 10 numbers. Over half of the number had one or even more than one control group. The follow-up time also increased from a few months to more than 1 year in most of studies. All of the results reported positive results for clinical bone regeneration.

## Conclusions

Tissue engineering materials are currently available clinically as bone substitutes and can be used as scaffolds in combination with the bone-stimulating agents to expedite bone healing, which has made great progress comparing to a decade ago. Application of scaffolds in clinical field showed a good ability to facilitate bone repair and osteogenesis. However, significant challenges still exist in clinical studies due to limitations and translational difficulties which prevent their implementation into clinical practice [51]. Currently, application of scaffolds on clinical field showed a good ability to facilitate bone repair and osteogenesis in our systematic review. This systematic review provided an ideal and reliable result for the further progression and development of clinical study, which will promote other researchers and readers in this tissue engineering fields to comprehensively understand the clinical results of scaffolds for bone regeneration and applied these achievements for the further clinical practice. In addition, the ideal and reliable guidelines need to be sufficiently applied and the number and quality of studies in this field remain need to be improved.

## Abbreviations

BMAC: Bone marrow aspiration concentrate
BMMNCs: Bone-marrow-derived mononuclear cells
CaPs: Calcium phosphate
CT: Computed tomography
IM: Induced membrane
IP-CHA: Interconnected porous calcium hydroxyapatite
MSC: Mesenchymal stromal cell
OHS: Oxford hip score
P: Periosteum
PRP: Platelet-rich plasma
rCPBS: Resorbable calcium phosphate bone substitute
rhBMP-2: Recombinant human bone morphogenetic protein-2
rhBMP-7: Recombinant bone morphogenetic protein 7
SAPS: Self-reported satisfaction scale
SF12: Short-form
TSRH: Texas Scottish Rite Hospital
